# Racial and Ethnic Disparities in the Diagnostic Performance of AI Systems for Medical Imaging in the United States: A Systematic Review

**DOI:** 10.7759/cureus.114001

**Published:** 2026-08-05

**Authors:** Gift Ojukwu, Chidiogo J Mamah, Steve Okwu, Akinyele Oladimeji, Ibrahim O Balogun, Obianuju M Odok

**Affiliations:** 1 General Practice, Leeds Teaching Hospitals NHS Trust, Leeds, GBR; 2 Radiology, Afe Babalola University Multi-system Hospital, Ado Ekiti, NGA; 3 Cardiothoracic Surgery, Beacon Hospital, Dublin, IRL; 4 Family Medicine, Alberta Health Services, Edmonton, CAN; 5 Internal Medicine, Bridgeport Hospital, Bridgeport, USA; 6 Radiology, University of Calabar, Calabar, NGA

**Keywords:** artificial intelligence, diagnostic performance, healthcare disparities, racial bias, radiology, systematic review

## Abstract

AI-assisted diagnostic systems are increasingly being integrated into radiology and medical imaging practice in the United States. Concerns have emerged regarding differences in diagnostic performance across racial and ethnic populations, particularly among historically underserved groups. This study aimed to systematically review evidence on racial and ethnic disparities in the diagnostic performance of AI systems used in radiology and medical imaging in the United States. A systematic search of major databases identified 27 eligible studies published between 2015 and 2026. The included studies primarily consisted of retrospective observational and validation studies evaluating AI systems in chest radiography, breast ultrasound, and ophthalmologic imaging, as well as selected non-imaging predictive models. Several studies reported reduced diagnostic performance, lower sensitivity, higher rates of underdiagnosis, or subgroup disparities among racial and ethnic minority populations, particularly Black and Hispanic patients. Some studies also demonstrated that fairness-aware model development and subgroup validation may improve equity in AI performance. The findings were synthesized narratively and support the need for diverse training datasets, transparent demographic reporting, standardized fairness evaluations, and prospective multicenter validation before the widespread clinical implementation of AI systems in radiology and medical imaging.

## Introduction and background

Rapid advancements in AI technologies have enabled the analysis of large volumes of medical imaging data with high efficiency and promising diagnostic performance [1]. Many medical devices incorporating AI algorithms, including computer-assisted diagnostic systems, have received clearance from the U.S. FDA for clinical use in radiology [2,3]. These technologies are increasingly being integrated into clinical practice to assist with image interpretation, disease detection, workflow optimization, and diagnostic decision-making [4]. AI applications have demonstrated promising performance in detecting breast cancer, lung disease, stroke, fractures, cardiovascular disease, and neurological disorders using medical imaging [5]. Consequently, AI has been viewed as a valuable tool for improving diagnostic accuracy, reducing human error, and enhancing the efficiency of radiology services [6].

Despite these advances, including the increasing availability of FDA-cleared AI tools for clinical use, concerns have emerged regarding whether AI systems used in radiology and medical imaging perform equitably across diverse racial and ethnic populations in the United States [7]. The performance of AI models depends heavily on the quality and representativeness of the datasets used during model development [8]. When racial and ethnic minority populations are underrepresented in training or validation datasets, AI systems may demonstrate reduced diagnostic accuracy or inconsistent performance in these groups compared with majority populations [9,10]. Such disparities have the potential to contribute to delayed diagnosis, inequitable clinical decision-making, and the widening of healthcare disparities among historically underserved racial and ethnic populations [11].

These concerns are particularly important in radiology, where racial and ethnic disparities in access to imaging services, disease outcomes, and diagnostic evaluation have been well documented [12,13]. Without adequate evaluation across diverse populations, AI tools may unintentionally perpetuate or amplify existing inequities in diagnostic performance [14,15]. Consequently, fairness, transparency, accountability, and equitable implementation have become central considerations in the development and deployment of AI-enabled medical imaging technologies [16].

In response, regulatory agencies, researchers, and professional organizations have emphasized the importance of evaluating AI systems across diverse patient populations before widespread clinical implementation [17]. Although many AI systems, including those that have received U.S. FDA clearance, are reported to have high overall diagnostic accuracy, uncertainty remains regarding whether this performance is consistent across racial and ethnic groups [18]. Individual studies have reported reduced sensitivity, specificity, or predictive performance among minority populations; however, the evidence remains dispersed across different imaging modalities and clinical applications [19].

To our knowledge, no previous systematic review has specifically synthesized evidence on racial and ethnic disparities in the diagnostic performance of AI systems used in radiology and medical imaging in the United States. This review addresses that gap by systematically identifying and narratively synthesizing the available evidence, with a particular focus on differences in diagnostic performance across racial and ethnic populations. The findings are intended to inform future AI development, validation, regulatory evaluation, and equitable implementation in radiology practice.

## Review

Materials and methods

Eligibility Criteria and Search Strategies

This systematic review was conducted and reported in accordance with the Preferred Reporting Items for Systematic Reviews and Meta-Analyses (PRISMA) 2020 guidelines to ensure methodological transparency and reproducibility [20]. The review question and search strategy were developed using the Population, Intervention, Comparison, Outcome (PICO) framework [21]. The PICO elements were defined as follows: Population: patients or populations evaluated in studies of AI used in radiology, medical imaging, or related diagnostic healthcare applications in the United States; Intervention: AI systems used for diagnostic imaging, clinical prediction, or decision support; Comparison: diagnostic or predictive performance across racial and ethnic populations; and Outcome: differences in diagnostic accuracy, sensitivity, specificity, predictive performance, fairness, validation, or other measures of algorithm performance.

Peer-reviewed studies published in English between 2015 and 2026 were eligible for inclusion. Studies were included if they evaluated AI systems used in radiology, medical imaging, or related diagnostic healthcare applications and reported findings relevant to racial or ethnic differences in algorithm performance, diagnostic accuracy, fairness, validation, implementation, or demographic reporting. Eligible study designs included retrospective and prospective observational studies, validation studies, algorithm evaluation studies, randomized controlled trials, systematic reviews and meta-analyses, narrative reviews, observational reviews, and scoping reviews, provided that they contributed evidence relevant to the review objectives.

Studies were excluded if they were conference abstracts, editorials, letters, commentaries without original or synthesized evidence, non-English publications, or studies that did not evaluate AI performance or report findings related to racial or ethnic disparities, fairness, validation, or diagnostic performance relevant to the review objectives.

A systematic literature search was conducted in PubMed, Scopus, Web of Science, Embase, and the Cochrane Library using combinations of controlled vocabulary terms, including Medical Subject Headings (MeSH), and keywords such as “Artificial Intelligence,” “Radiology,” “Medical Imaging,” “Diagnostic Performance,” “Racial Disparity,” “Ethnic Disparity,” and “Algorithmic Bias.” Titles, abstracts, and full-text articles were screened against the predefined eligibility criteria, and the reference lists of eligible studies were manually searched to identify additional relevant publications.

Table 1 provides an overview of the literature search strategy, including the databases searched, time frame, and key search terms.

**Table 1:** Overview of the search strategy.

Category	Details
Databases Searched	PubMed, Scopus, Web of Science, Embase, and the Cochrane Library
Time Frame	2015-2026
Language	English only
Search Strategy Structure	#1 AND #2 AND #3
#1 (Population/Setting)	“medical imaging” OR “radiology” OR “diagnostic imaging” OR “radiologic imaging”
#2 (Intervention)	“artificial intelligence” OR “machine learning” OR “deep learning” OR “AI-based diagnostic tools” OR “clinical prediction models”
#3 (Outcome/Equity)	“racial disparities” OR “ethnic disparities” OR “diagnostic performance” OR “sensitivity” OR “specificity” OR “predictive accuracy” OR “algorithmic bias” OR “fairness”

Inclusion Criteria

Studies were eligible for inclusion if they evaluated AI systems used in radiology, medical imaging, or related diagnostic healthcare applications and reported findings relevant to racial or ethnic differences in diagnostic or predictive performance, fairness, validation, implementation, or demographic reporting. Studies involving adult populations undergoing medical imaging were eligible, as were selected studies of diagnostic or predictive healthcare AI applications relevant to the review objectives. Study designs included randomized controlled trials, retrospective and prospective observational studies, validation studies, algorithm evaluation studies, systematic reviews and meta-analyses, narrative reviews, observational reviews, and scoping reviews published in English between 2015 and 2026. Eligible studies reported outcomes related to diagnostic accuracy, sensitivity, specificity, area under the receiver operating characteristic curve (AUC), predictive performance, algorithm fairness, or other measures of AI performance across racial and ethnic populations.

Exclusion Criteria

Conference abstracts; editorials; commentaries; letters to the editor; narrative reviews; case reports; laboratory studies; animal studies; non-peer-reviewed articles; studies conducted outside the United States; studies not evaluating FDA-cleared AI tools in radiology; articles that did not report diagnostic test outcomes stratified by racial or ethnic subgroup; articles that did not report diagnostic test outcomes; studies conducted exclusively among pediatric patients; and studies evaluating non-imaging AI applications were excluded.

Screening Process

All identified records were loaded into EndNote, and duplicates were removed. Study eligibility was determined by two independent reviewers who screened the titles and abstracts against the predefined eligibility criteria. Full-text assessments of potentially relevant studies were then conducted independently by both reviewers. Disagreements were resolved through discussion or, when consensus could not be reached, through the involvement of an additional reviewer, thereby ensuring methodological rigor and consistency.

Quality Assessment

Methodological quality and risk of bias were evaluated using validated quality assessment instruments. Randomized controlled trials were assessed using the Cochrane Risk of Bias 2 (RoB 2) tool [22], whereas the Newcastle-Ottawa Scale (NOS) [23] was used as a standardized framework to evaluate the methodological quality of the remaining included studies. Although the review included studies with heterogeneous designs, including observational studies, reviews, and implementation or governance studies, the NOS was applied consistently to provide a uniform assessment of methodological quality based on study selection, comparability, and outcome reporting. The resulting quality ratings should therefore be interpreted in the context of the diverse study designs included in this narrative systematic review.

Data Extraction

A standardized data extraction form was created to collect key study and demographic information, including author name, publication year, study design, sample size, imaging modality, type of FDA-cleared AI tool evaluated, patient demographics, and racial and ethnic subgroup distribution, as well as diagnostic performance measures such as sensitivity, specificity, AUC, and predictive accuracy.

Data Analysis

Data synthesis was conducted using a qualitative narrative approach. Studies included in the systematic review were categorized according to clinical application, imaging modality, and type of AI system evaluated. A narrative synthesis was performed to summarize the study characteristics, methodological approaches, and reported findings related to racial and ethnic differences in AI diagnostic performance.

The findings of the included studies were reviewed and compared descriptively, with emphasis placed on reported disparities in diagnostic performance, underdiagnosis, predictive accuracy, fairness concerns, and subgroup differences across racial and ethnic populations. Attention was also given to variations in study design, patient populations, AI algorithms, and clinical settings.

Due to substantial methodological and clinical variability across the included studies, the findings were synthesized narratively to provide a comprehensive overview of the existing evidence on racial and ethnic bias in healthcare AI systems.

Results

**Figure 1:**
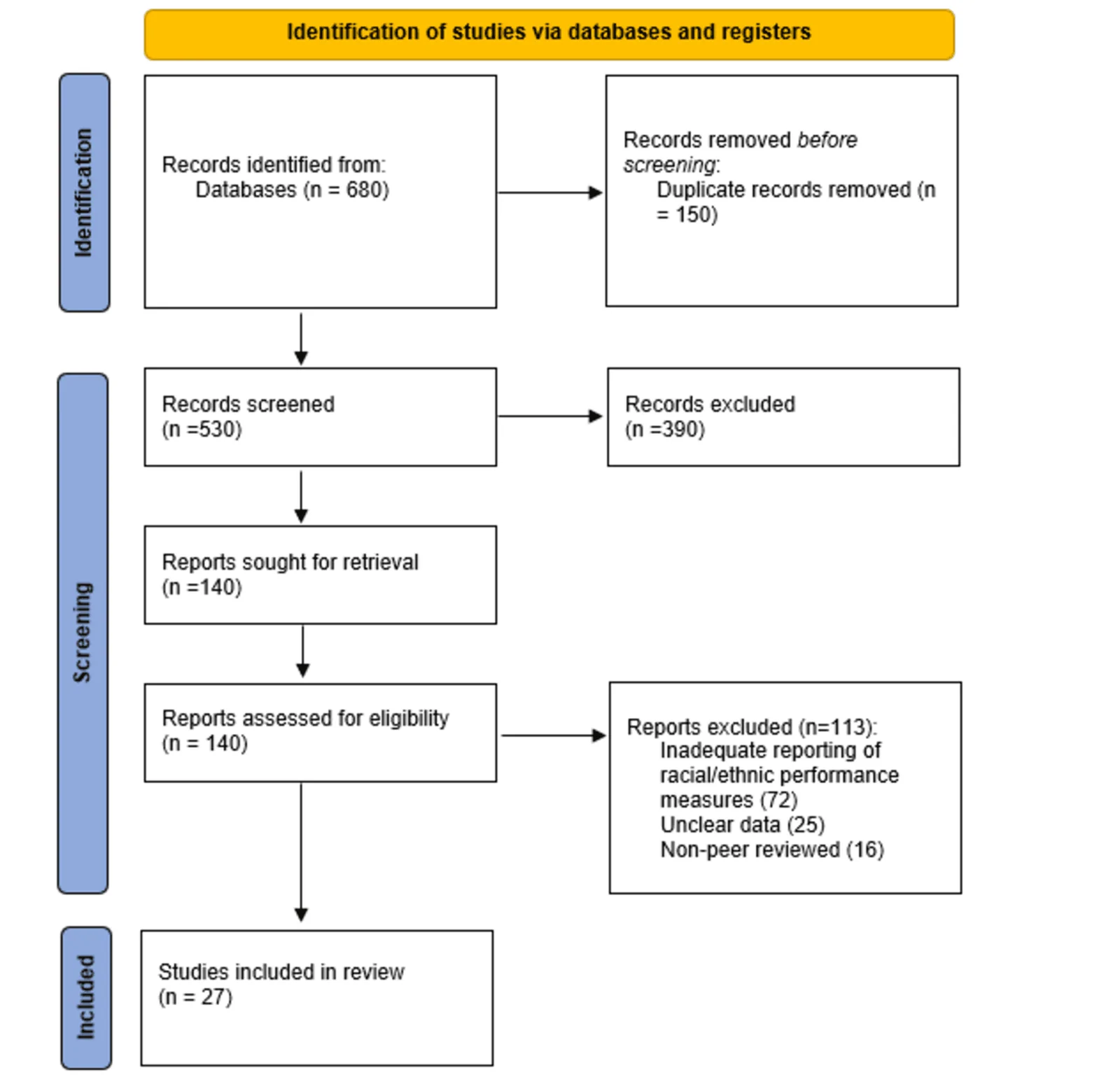
PRISMA flow diagram showing the study selection and inclusion process. This review was reported in accordance with the PRISMA 2020 guidelines [20]. The figure was adapted from the PRISMA 2020 flow diagram template, which is licensed under the Creative Commons Attribution 4.0 International (CC BY 4.0) license. PRISMA: Preferred Reporting Items for Systematic Reviews and Meta-Analyses.

The data synthesis identified 680 records through the electronic literature search. Of these, 150 duplicates were removed, leaving 530 titles and abstracts for relevance screening. Of the 530 records screened, 390 were excluded because they did not meet the inclusion criteria, did not evaluate an FDA-cleared AI tool in radiology, did not include race- and/or ethnicity-specific subgroup analyses, or did not report diagnostic performance outcomes, such as sensitivity, specificity, or predictive accuracy. Following a detailed review of the 140 full-text articles, 113 articles were excluded for the following reasons: inadequate reporting of racial and/or ethnic performance measures, non-U.S. study populations, pediatric-only populations, evaluation of AI in non-imaging applications, and ineligible publication types, including conference abstracts, editorials, and narrative reviews. Ultimately, 27 studies met the predefined eligibility criteria and were included in the systematic review (Figure 1). The characteristics of the included studies are presented in Table 2.

**Table 2:** Summary of the included studies.

Reference	Study Design	Population	Outcome Focus	Key Findings
Liu X et al. (2019) [1]	Systematic review and meta-analysis	Patients undergoing medical imaging	AI diagnostic accuracy compared with clinicians	Deep learning models achieved diagnostic performance comparable to that of healthcare professionals across different imaging modalities.
Berson ER et al. (2023) [2]	Narrative observational review	Neuroimaging and musculoskeletal radiology populations	Commercial AI algorithms in radiology	The review noted that the FDA had cleared many artificial intelligence (AI) tools for clinical use and that their use in radiology was growing rapidly.
van Leeuwen KG et al. (2021) [3]	Observational review	Commercial radiology AI products	Scientific validation evidence	The review found that commercial radiology AI products required better external validation and reporting of patient demographics.
Choy G et al. (2018) [4]	Observational review	Radiology imaging populations	Machine learning applications in radiology	Machine learning could be applied broadly to improve imaging diagnosis, optimize radiology workflows, and perform predictive analytics.
Ueda D et al. (2021) [5]	Retrospective validation study	Patients undergoing chest radiography	AI-assisted lung cancer detection	AI assistance improved radiologists’ ability to detect lung cancer across multiple facilities.
Harada Y et al. (2021) [6]	Randomized controlled trial	Physicians using AI-assisted diagnosis	Differential diagnostic accuracy	The use of AI-generated differential diagnoses improved physicians’ diagnostic reasoning.
Seyyed-Kalantari L et al. (2021) [7]	Observational model-evaluation study	Historically underserved patient populations	Underdiagnosis bias in chest radiography AI	Lower diagnostic performance was documented for AI algorithms applied to historically underserved and minority populations.
Duffy G et al. (2022) [8]	Observational study	Medical imaging datasets	AI prediction of demographic characteristics	AI systems identified demographic characteristics and potential confounders in imaging data, raising concerns about fairness.
Lin M et al. (2023) [9]	Observational study	Patients with glaucoma	Diagnostic bias in deep learning models	Deep learning models demonstrated underdiagnosis and overdiagnosis biases among historically underserved populations.
Allen A et al. (2020) [10]	Observational algorithm-development study	Hospitalized patient cohorts	Fairness in mortality prediction	Racial equity in mortality prediction models could be improved by implementing bias-reduction measures.
Wang N et al. (2023) [11]	Observational study	Emergency department populations	Health information technology and racial disparities	Greater engagement with digital health helped reduce racial disparities in preventable emergency department visits.
Ross AB et al. (2020) [12]	Observational study	U.S. emergency department patients	Racial disparities in diagnostic imaging	Black and Hispanic patients were less likely to receive imaging than their White counterparts.
Johnson TJ et al. (2022) [13]	Retrospective observational study	Pediatric emergency department patients	Wait-time disparities	Minority patients had longer emergency department wait times before receiving care.
Banerjee I et al. (2023) [14]	Observational evaluation study	Radiology AI systems	Bias resulting from shortcut learning	AI systems learned spurious associations that contributed to racial and demographic biases in medical imaging.
Ricci Lara MA et al. (2022) [15]	Observational fairness study	Medical imaging AI datasets	AI fairness evaluation	The study summarized methods for evaluating and reducing bias in medical imaging AI.
Liao F et al. (2022) [16]	Observational implementation study	Large U.S. health system	Governance of clinical AI	The report described frameworks for accountability, transparency, and the equitable deployment of AI systems.
Larrazabal AJ et al. (2020) [17]	Observational model-evaluation study	Medical imaging datasets	Dataset imbalance and bias	Demographic imbalance in the datasets used to develop AI diagnostic classifiers resulted in biased outcomes.
Koo C et al. (2023) [18]	Retrospective observational study	Breast ultrasound imaging populations	Racial and ethnic AI performance	The racial and ethnic fairness of AI decision-support systems was evaluated using breast ultrasound.
Sun J et al. (2022) [19]	Prospective observational study	Patients undergoing chest radiography for COVID-19	AI diagnostic performance	Subgroup differences were identified in the accuracy of chest radiography AI tools.
Schut RA et al. (2020) [24]	Observational cohort study	Patients with incidental pulmonary nodules	Disparities in follow-up adherence	Minority patients had lower adherence to recommended imaging follow-up.
Wang Y et al. (2024) [25]	Observational fairness study	Patients with chronic diseases	Racial bias in mortality prediction	Evaluation of predictive performance identified significant racial disparities in machine learning models.
Khunte M et al. (2023) [26]	Retrospective observational study	FDA-cleared AI imaging algorithms	Clinical validation trends	Many FDA-cleared AI tools lacked reporting on demographic diversity in their validation datasets.
Akinrinmade AO et al. (2023) [27]	Observational review	Healthcare AI systems	Perceptions and implementation of AI	The review discussed discrepancies between expectations of AI and the challenges associated with its real-world implementation in healthcare.
Muralidharan V et al. (2024) [28]	Scoping review	FDA-authorized AI medical devices	Reporting gaps in AI validation	The review identified insufficient racial and ethnic reporting in studies of FDA-authorized AI medical devices.
Abashidze N et al. (2021) [29]	Observational cohort study	Patients undergoing prostate MRI	Disparities in imaging utilization	Black and Hispanic patients had lower utilization of prostate MRI following elevated prostate-specific antigen test results.
Kocher MR et al. (2022) [30]	Observational perspective analysis	Medical imaging AI systems	Prevention of AI bias	The study highlighted the risk of perpetuating healthcare disparities through biased AI systems.
Topiwala R et al. (2019) [31]	Retrospective observational study	Patients evaluated for sepsis	Machine learning diagnostic performance	Machine learning approaches improved the early identification of sepsis in clinical settings.

Among the 27 studies included in the systematic review, the majority consisted of retrospective and prospective observational cohort studies evaluating AI systems across multiple clinical applications and imaging modalities, including chest radiography, breast ultrasound, ophthalmologic imaging, and electronic health record-based predictive models. Across the reviewed literature, diagnostic performance varied considerably according to the AI model used, imaging modality, study population, and outcome definitions. Several studies reported differences in diagnostic accuracy across racial and ethnic groups, with minority populations in some settings experiencing lower sensitivity, reduced predictive performance, higher rates of underdiagnosis, or disparities in clinical risk prediction. Due to substantial variability in study designs, AI methodologies, fairness assessment approaches, and reporting standards, the findings were synthesized using a narrative approach to provide a comprehensive overview of the current evidence regarding racial and ethnic bias in healthcare AI systems.

Table 3 presents the methodological quality assessment of the included observational studies using the NOS [21]. Given the heterogeneity of the included study designs, the quality assessment scores should be interpreted as an overall indication of methodological rigor rather than as directly comparable measures across different study types.

**Table 3:** Quality assessment of observational studies using the Newcastle-Ottawa Scale. The Newcastle-Ottawa Scale (NOS) has a maximum score of nine stars: up to four for selection, two for comparability, and three for outcome assessment. The NOS is a freely available tool for assessing the methodological quality of nonrandomized observational studies and was applied in accordance with published guidance [23].

Study (First Author and Year)	Selection (Max 4)	Comparability (Max 2)	Outcome (Max 3)	Total Score (Max 9)	Quality Rating
Liu X et al. (2019) [1]	4	2	3	9	High
Berson ER et al. (2023) [2]	3	1	2	6	Moderate
van Leeuwen KG et al. (2021) [3]	4	2	3	9	High
Choy G et al. (2018) [4]	3	1	2	6	Moderate
Ueda D et al. (2021) [5]	4	2	3	9	High
Seyyed-Kalantari L et al. (2021) [7]	4	2	3	9	High
Duffy G et al. (2022) [8]	4	2	2	8	High
Lin M et al. (2023) [9]	3	2	2	7	Moderate
Allen A et al. (2020) [10]	4	2	3	9	High
Wang N et al. (2023) [11]	3	2	2	7	Moderate
Ross AB et al. (2020) [12]	4	2	3	9	High
Johnson TJ et al. (2022) [13]	4	2	2	8	High
Banerjee I et al. (2023) [14]	3	2	2	7	Moderate
Ricci Lara MA et al. (2022) [15]	4	2	3	9	High
Liao F et al. (2022) [16]	3	2	2	7	Moderate
Larrazabal AJ et al. (2020) [17]	4	2	3	9	High
Koo C et al. (2023) [18]	4	2	3	9	High
Sun J et al. (2022) [19]	4	2	3	9	High
Schut RA et al. (2020) [24]	4	2	2	8	High
Wang Y et al. (2024) [25]	4	2	3	9	High
Khunte M et al. (2023) [26]	3	2	2	7	Moderate
Akinrinmade AO et al. (2023) [27]	3	1	2	6	Moderate
Muralidharan V et al. (2024) [28]	4	2	3	9	High
Abashidze N et al. (2021) [29]	4	2	3	9	High
Kocher MR et al. (2022) [30]	3	1	2	6	Moderate
Topiwala R et al. (2019) [31]	4	2	3	9	High

Table 4 shows the risk-of-bias assessment of the included randomized controlled trial using the Cochrane RoB 2 tool [22].

**Table 4:** Risk-of-bias assessment of the randomized controlled trial using the RoB 2 tool. The Cochrane Risk of Bias 2 (RoB 2) tool is freely available and was applied in accordance with established guidance for randomized trials [22].

Study (First Author and Year)	Randomization Process	Deviations from Intended Interventions	Missing Outcome Data	Measurement of the Outcome	Selection of the Reported Result	Overall Risk of Bias
Harada Y et al. (2021) [6]	Low	Low	Low	Low	Low	Low

Study Findings

The systematic review showed that although AI tools cleared by the U.S. FDA often demonstrate high overall diagnostic or prognostic accuracy when applied to medical images, substantial differences in performance have been reported across racial and ethnic groups, particularly among minority and historically underserved populations [1,7,18].

Most observational and validation studies concluded that AI systems trained on imbalanced data were more likely to exhibit higher underdiagnosis rates, lower sensitivity, or lower positive predictive values for non-White patients than for White patients [7,9]. For example, Seyyed-Kalantari L et al. [7] identified a significant degree of underdiagnosis bias in AI algorithms used to interpret chest radiographs among historically underserved populations. Similarly, Lin M et al. [9] identified underdiagnosis and overdiagnosis biases in AI algorithms used to diagnose glaucoma.

Several studies indicated that inadequate demographic representation during model development and validation can result in AI algorithms exhibiting demographic biases. For example, Larrazabal AJ et al. [17], Duffy G et al. [8], and Banerjee I et al. [14] indicated that AI systems can learn spurious associations involving demographic characteristics, including race and ethnicity, when imaging datasets are imbalanced or contain unrecognized confounders.

Wang Y et al. [25] provided empirical evidence of racial bias in machine learning models developed to predict mortality, highlighting the need for a validation process to evaluate algorithmic fairness. Moreover, Khunte M et al. [26] found that many FDA-cleared AI tools in the imaging domain did not provide adequate information regarding their clinical validation. Finally, Akinrinmade AO et al. [27] discussed the contrast between the perceived benefits of AI in healthcare and the practical challenges encountered when attempting to implement AI fairly and transparently.

A substantial body of research demonstrated that racial and ethnic disparities can affect access to imaging examinations. Several retrospective cohort and implementation studies documented that racial and ethnic minority patients were less likely to receive diagnostic imaging. Studies focusing on governance also addressed the need to evaluate fairness and outcomes when widely implementing FDA-cleared AI systems in clinical practice [15,16,26,28]. For example, Ross AB et al. [12] and Abashidze N et al. [29] found that racial and ethnic minority patients had reduced access to diagnostic imaging and prostate MRI. In addition, Schut RA et al. [24] found that non-White patients were less likely than White patients to adhere to follow-up recommendations after an incidental pulmonary nodule was detected.

Overall, the findings suggest that AI tools used in radiology may perpetuate or exacerbate healthcare inequities unless demographic diversity, subgroup validation, and fairness assessments are incorporated into their development, validation, and clinical deployment.

Discussion

Racial and Ethnic Disparities in the Diagnostic Performance of FDA-Cleared AI Tools in Radiology

The studies included in this review indicate that several AI systems used in radiology and related diagnostic applications demonstrated differences in diagnostic performance across racial and ethnic populations, particularly among historically underrepresented groups. However, the magnitude and direction of these differences varied across imaging modalities, AI models, and study populations. Although many AI algorithms demonstrated high overall diagnostic accuracy, subgroup analyses in several studies reported lower sensitivity, reduced predictive performance, or higher rates of underdiagnosis among Black, Hispanic, and other racial and ethnic minority populations [1,7,18].

Several studies attributed these disparities, at least in part, to the underrepresentation of demographic groups, including racial and ethnic minority populations, in training and validation datasets, potentially limiting model generalizability across diverse clinical settings [7,17]. Studies of chest radiography, breast ultrasonography, glaucoma screening, and mortality prediction showed measurable evidence of underdiagnosis and overdiagnosis biases among non-White patient populations [7,9,25].

Whether through hidden confounding variables or “shortcut learning” mechanisms within large medical imaging datasets, AI-based systems may infer an individual’s race or other demographic characteristics from medical images, creating the potential for biased predictions and inequitable healthcare delivery [8,14]. Collectively, these findings highlight concerns regarding dataset diversity, subgroup validation, and the generalizability of AI systems across diverse populations [15,30].

Representation, Validation, and Fairness Assessment in FDA-Cleared AI Systems

The review also identified important gaps in demographic reporting, subgroup validation, and fairness evaluation during the development and validation of AI systems used in radiology and related diagnostic applications [3,26,28]. Several studies recommended incorporating fairness assessments, standardized demographic reporting, and external validation into AI development and evaluation to improve transparency and support equitable implementation [31]. In addition, researchers who explored governance issues related to the ethical deployment of AI recommended creating standardized reporting frameworks, auditing for bias, and conducting ongoing postmarketing surveillance of AI products in real-world practice to identify and address differences in performance across demographic groups [15,16].

To ensure the equitable implementation of AI products, researchers strongly recommended using multicenter datasets that adequately represent underserved communities to train AI algorithms and conducting subgroup validation before adopting AI products into clinical practice [14,26]. Collectively, the reviewed studies suggest that broader demographic representation, external validation, and routine subgroup evaluation may improve the equitable implementation of AI systems in clinical practice.

Healthcare Access, Imaging Utilization, and Clinical Implications of AI Bias

Beyond algorithm performance, studies included in the review demonstrated the continued existence of racial and ethnic disparities in access to diagnostic imaging and follow-up care within the U.S. healthcare system. More specifically, racial and ethnic minority patients, particularly Black patients, were less likely than White patients to receive diagnostic imaging [12].

A lack of timely access to imaging, as well as limited adherence to established follow-up recommendations, may exacerbate the potential harms associated with biased AI systems when these systems are used in already underserved healthcare settings [24]. Studies investigating healthcare technology and AI implementation found that structural inequities, disparities in healthcare infrastructure, and unequal levels of digital engagement affected the effectiveness of AI-based clinical tools in practice [11,27].

Additionally, observational studies indicate that biased predictive models could contribute to unequal risk stratification and clinical decision-making, potentially leading to disparities in preventable morbidity and mortality among minority patients [10,25]. Thus, achieving equitable implementation of AI in radiology requires both fair algorithm development and broader efforts to reduce systemic disparities related to healthcare access, imaging utilization, and clinical follow-up.

Implications for Clinical Implementation

The reviewed studies identified several challenges that may influence the implementation of AI in clinical practice. These included limited external validation of commercially available AI systems, insufficient demographic reporting, and the predominance of retrospective observational evidence supporting many AI applications [3,11,26,28]. Several studies also highlighted the importance of transparent governance, accountability, clinician education, and ongoing evaluation to support the safe and equitable adoption of AI in radiology [4,15,16]. In addition, successful implementation will require appropriate workforce training, integration into existing clinical workflows, and continued assessment of AI performance across diverse patient populations to ensure equitable clinical use [16,27,32,33].

Limitations

This systematic review has several limitations that should be acknowledged. Most included studies were retrospective observational or validation studies, which may limit the strength of the available evidence and the generalizability of the findings across different healthcare settings and populations. Considerable methodological and conceptual heterogeneity existed among the included studies with respect to study design, AI applications, imaging modalities, patient populations, clinical settings, fairness assessment methods, and outcome measures, making direct comparisons across studies challenging.

In addition, although the review focused on racial and ethnic disparities in AI used in radiology and related diagnostic healthcare applications, the included studies varied in scope and included evidence beyond FDA-cleared radiology AI tools. Consequently, the findings should be interpreted as a broad synthesis of the available evidence rather than as conclusions applicable exclusively to FDA-cleared radiology AI systems.

Many studies also lacked standardized reporting of racial and ethnic subgroup outcomes and did not provide sufficiently detailed quantitative diagnostic performance data. This precluded a formal meta-analysis and limited consistent quantitative comparisons across studies. Variability in fairness assessment methods and reporting practices further complicated the interpretation of the findings.

Finally, only English-language articles published between 2015 and 2026 were included, which may have resulted in the exclusion of relevant studies published in other languages or outside the specified time frame. Publication bias and the selective reporting of favorable AI performance may also have influenced the overall conclusions of this review.

## Conclusions

This systematic review suggests that racial and ethnic differences in the diagnostic performance of AI systems used in radiology and medical imaging have been reported in several studies conducted in the United States. Although many AI systems demonstrated high overall diagnostic performance, some studies identified reduced accuracy, lower sensitivity, or higher rates of underdiagnosis among racial and ethnic minority populations. Given the methodological and conceptual heterogeneity of the included literature, these findings should be interpreted cautiously. Nevertheless, this review highlights the importance of representative training datasets, subgroup validation, and transparent demographic reporting to support the equitable evaluation and implementation of AI systems in radiology and medical imaging. Future multicenter prospective studies using standardized fairness assessment and reporting frameworks are needed to strengthen the evidence base and improve understanding of AI performance across diverse populations.
